# The Impact of Anodal tDCS on Verbal and Nonverbal Functional Communication in Subacute Aphasia—An Observational Study

**DOI:** 10.3390/brainsci16070727

**Published:** 2026-07-09

**Authors:** Ilona Rubi-Fessen, Kathrin Gerbershagen, Prisca Stenneken, Klaus Willmes

**Affiliations:** 1 Neurological Rehabilitation Hospital, RehaNova Köln, 51109 Cologne, Germany; gerbershagen@rehanova.de; 2 Department of Rehabilitation and Special Education, Faculty of Human Sciences, University of Cologne, 50931 Cologne, Germany; prisca.stenneken@uni-koeln.de; 3 Department of Neurology, Medical Faculty, RWTH Aachen University, 52074 Aachen, Germany; willmes@neuropsych.rwth-aachen.de

**Keywords:** subacute aphasia, verbal and nonverbal functional communication, non-invasive brain stimulation (NIBS), transcranial direct current stimulation (tDCS), aphasia therapy, language

## Abstract

**Highlights:**

**What are the main findings?**

- Adjuvant anodal tDCS over preserved left-hemispheric language areas produced significantly greater improvements in verbal functional communication (measured by the Amsterdam–Nijmegen Everyday Language Test; ANELT) and nonverbal functional communication (measured by the Scenario Test) during tDCS-supported therapy than during speech and language therapy alone in individuals with subacute aphasia.
- With regard to nonverbal communication, individuals with severe subacute aphasia used various and different channels to convey communicative messages in the course of rehabilitation.

**What are the implications of the main findings?**

- Individually tailored anodal tDCS is a feasible, well-tolerated, and clinically promising adjuvant tool for enhancing functional communicative outcomes—including nonverbal communication—in early aphasia rehabilitation, supporting its integration into routine clinical practice.
- The parallel assessment of linguistic performance and functional communicative competence using standardized, performance-based instruments that capture both verbal and nonverbal communication should become standard practice in aphasia rehabilitation research and clinical diagnostics, as linguistic measures alone are insufficient to capture the full range of therapy-induced recovery.

**Abstract:**

**Background/Objectives**: While a growing number of studies have demonstrated positive effects of adjuvant anodal transcranial direct current stimulation (tDCS) on linguistic performance in aphasia, evidence for corresponding effects on verbal and—in particular—nonverbal functional communication remains absent. This is a critical gap, given that restoring everyday communicative competence is the ultimate goal of speech and language therapy (SLT). In the present observational study, we investigated the add-on effect of anodal tDCS over language-relevant left-hemispheric areas on verbal and nonverbal functional communication in n = 34 individuals with subacute aphasia and examined the relationship between communicative and linguistic change. **Methods**: Participants underwent two consecutive two-week therapy phases (P1, P2), each consisting of 10 SLT sessions. Severity-specific linguistic and communicative assessments were administered before, between, and after both phases: the Aachen Aphasia Test (AAT; n = 26) combined with the Amsterdam–Nijmegen Everyday Language Test (ANELT, A-scale), and the Bielefeld Aphasia Screening Rehabilitation (BIAS-R; n = 8) combined with the Scenario Test. During P2, SLT was supplemented by online anodal tDCS. **Results**: Overall test performance improved significantly more in tDCS-supported P2 compared to P1 (AAT profile level, *p* < 0.001; BIAS-R mean percentage value (MPW), *p* = 0.027; ANELT A-scale raw score Version 1, *p* = 0.081, and version 2, *p* = 0.038; and Scenario Test total score, *p* = 0.003). Significant correlations between AAT profile level and ANELT total scores were found across all time points. Between the MPV and subtests of the BIAS-R and the Scenario total score, there was a tendency toward decreasing correlation levels from T1 to T3. **Conclusions:** To our knowledge, this is the first study demonstrating that adjuvant tDCS in subacute aphasia enhances not only linguistic performance but also verbal and nonverbal functional communication beyond SLT alone—assessed with standardized, performance-based, ecologically valid instruments.

## 1. Introduction

Aphasia is one of the most devastating and socially disabling consequences of stroke. In the acute phase, aphasia affects approximately 30–40% of stroke survivors, with prevalence estimates varying across studies depending on assessment timing [1,2,3]. As an acquired language disorder, aphasia impairs verbal expression, auditory comprehension, reading, and writing to varying degrees.

However, the impact of aphasia extends well beyond the language system itself. Both verbal and nonverbal communication are fundamental to virtually every aspect of daily life: they enable people to express their needs, emotions, and preferences; to maintain relationships; to engage in professional and civic life; and to exercise autonomy over decisions that affect their health and wellbeing. When verbal communicative competence is suddenly and severely restricted by aphasia, the consequences are wide-ranging. Since the loss of verbal communicative competence is also a fundamental challenge to self-determination, and participation in social life [4,5], people with aphasia (PwA) experience significantly heightened rates of depression, social isolation, and reduced quality of life (QoL) [6,7,8,9].

For this reason, the ultimate goal of speech and language therapy (SLT) in aphasia rehabilitation is not restricted to the restoration of impaired linguistic functions, but encompasses the recovery of functional, everyday communication in all its aspects [10,11,12]. This includes not only verbal communication, such as speaking and writing, but also the use of nonverbal communication channels such as gesturing, drawing, or pointing [13]. In many PwA, particularly those with severe or persistent aphasia, nonverbal communication strategies become critical compensatory resources that allow messages to be conveyed even when verbal output remains limited or absent [14,15,16].

Despite this consensus on the overarching goals of aphasia therapy, the evidence base for SLT—and especially for novel adjuvant approaches—has prioritized linguistic outcome measures such as naming accuracy, repetition, or composite aphasia test scores. Although assessment of therapy outcomes should reflect a multimodal communicative reality rather than focusing exclusively on linguistic performance measures [17,18], measures of functional communicative performance in ecologically valid, everyday contexts have received considerably less attention, particularly in the subacute phase of recovery. This is a significant gap, given that improvements in standardized language tests do not necessarily translate into gains in functional communication [12,19,20]. Speech and language therapy remains the gold standard intervention for aphasia across all stages of recovery [21,22]. For chronic aphasia, there is now robust evidence that intensive SLT produces meaningful linguistic and functional gains [22]. In the acute and subacute phases, however, the evidence is more limited and less consistent [19,23,24], in part because spontaneous recovery during this period makes it difficult to isolate treatment-related change from spontaneous remission.

One promising strategy for enhancing the efficacy of behavioural SLT is the adjuvant use of non-invasive brain stimulation (NIBS), in particular transcranial direct current stimulation (tDCS). tDCS is a safe, well-tolerated form of electrostimulation that modulates cortical excitability by delivering weak constant currents (typically 1–2 mA) to the scalp through surface electrodes and can be applied simultaneously with behavioural therapy—a so-called “online” stimulation paradigm [25,26,27]. There is converging evidence from systematic reviews and meta-analyses that anodal tDCS over the left hemisphere—particularly over the left inferior frontal gyrus (IFG)—combined with SLT can produce significant gains in linguistic performance in chronic aphasia [28,29,30,31,32,33].

Given that neuroplasticity is greatest in the early period after stroke, the acute and subacute phase could represent the optimal window for neuromodulatory interventions to support language recovery [34,35]. Yet only a small number of studies have examined the effects of tDCS as an adjuvant to SLT at this stage, and results have been inconsistent. Spielmann et al. [36] found no add-on effect of anodal tDCS over the left IFG combined with a word-finding treatment on naming or on verbal communication in 58 individuals with subacute aphasia, compared to sham stimulation. Stockbridge et al. [37], using a hierarchical, lesion-guided approach to stimulation site selection, found no significant effect on picture naming but did detect a positive effect on verbal functional communication measures in a cohort of 58 PwA. This difference between linguistic and communicative outcomes illustrates both the limited sensitivity of naming tasks to reflect everyday communicative change and the potential for tDCS to facilitate broader network-level effects that manifest more clearly at the communicative than at the impairment level.

In a recent observational study, we demonstrated for the first time that individually tailored anodal tDCS protocols targeting preserved left-hemispheric language tissue can be feasibly and safely implemented within the routine of an early rehabilitation clinic [38]. In a sample of 37 individuals with subacute aphasia, linguistic performance improved significantly more during the period of combined SLT and tDCS than during a preceding period of SLT alone. These findings held across both a moderate-to-mildly affected group assessed with the Aachen Aphasia Test (AAT) [39] and a more severely affected group assessed with the Bielefeld Aphasia Screening-Reha (BIAS-R) [40]. Importantly, however, the former analyses focused primarily on linguistic outcome measures.

As people with aphasia, particularly those with severe or persisting impairment, frequently rely on multimodal compensatory strategies including gesturing, drawing, and pointing to convey meaning when verbal output is unavailable or insufficient [13,17,41], any assessment framework that neglects nonverbal communicative channels therefore risks underestimating functional communicative competence, especially in more severely affected individuals. Furthermore, while verbal communication and overall functional communication skills are increasingly being assessed in studies on the effectiveness of tDCS in combination with aphasia therapy [36,37,42,43], or cognitive training [44], to our knowledge, the effect of additive tDCS on nonverbal communication has not yet been investigated or reported in any study. Furthermore, most studies assess functional communication with subjective measures completed by a proxy, like the American Speech-Language-Hearing Association Functional Assessment of Communication Skills for Adults (ASHA-FACS) [45] or the Communicative Effectiveness Index (CETI) [46]. The present study therefore extends this prior work by examining whether the add-on benefit of anodal tDCS observed for linguistic measures also generalizes to performance-based, ecologically validated measures of verbal and nonverbal functional communication. Beyond examining a possible communicative add-on effect of tDCS, the present analysis, which refers to the same sample of PwA as in Rubi-Fessen et al. [38], also aims at investigating the specific relationship between linguistic and communicative change across both therapy periods. Addressing these questions might have direct implications for the design of outcome assessments in future aphasia therapy trials and for the structuring of rehabilitation programs that aim to restore not only language, but also the full range of communicative participation that contributes strongly to quality of life after aphasia.

## 2. Materials and Methods

### 2.1. Study Design

The present study employed a within-subject, pre–post observational design consisting of two consecutive two-week treatment phases (P1 and P2), each comprising ten individual SLT sessions of 45 min duration. During P2, behavioural SLT was supplemented by online anodal tDCS (20 min, 2 mA) applied over the left hemisphere. Severity-specific linguistic assessments and assessments for verbal and nonverbal functional communication were conducted at three time points (T1–T3): T1—conducted on the weekday (Monday–Friday) before therapy phase P1; T2—conducted on the weekday after therapy phase P1 and the weekday before therapy phase P2; and T3—conducted on the weekday after therapy phase P2.

P1, consisting of SLT without brain stimulation, served as an intraindividual control condition against which the add-on effect of tDCS during P2 could be evaluated. Changes in linguistic and communicative performance were compared across the two therapy periods, allowing for both within-period and between-period analyses. The fixed sequence of therapy periods—P1 consisting of SLT alone followed by P2 combining SLT with anodal tDCS—and the deliberate decision against a crossover design was motivated by two considerations: first, the substantial difference in the influence of spontaneous remission between P1 and P2 arising from the partly early subacute stage of aphasia at study entry; and second, the impossibility of implementing an adequate washout period of at least two weeks between periods—which would have been required to preclude carry-over effects of tDCS administered in P1 on outcomes in P2—within the constraints of the limited inpatient rehabilitation stay.

### 2.2. Participants

Participants were recruited from the neurological rehabilitation clinic RehaNova Köln gGmbH (Cologne, Germany) between May 2019 and January 2024. Potential participants were identified and referred by the treating speech–language pathologist (SLP) or the attending physician.

Eligibility criteria included: unilateral left-hemispheric ischemic or hemorrhagic stroke affecting the middle cerebral artery territory; aphasia in the subacute phase as defined by Bernhardt et al. [47], spanning from day 7 to the end of month 6 post-onset (with the additional distinction of early subacute (day 7 to end of month 3), and late subacute (month 4 to end of month 6); confirmed right-handedness assessed via the Laterality Questionnaire by Salmaso and Longoni [48]; sufficient testability and capacity to follow simple verbal instructions; and an attention span lasting for sessions of about 60 to 90 min.

Participants were excluded if they had a prior history of cerebrovascular stroke with language impairment, a diagnosis of neurodegenerative or psychiatric disease, epileptiform EEG activity or a history of seizures, or sensory deficits (auditory or visual) that would preclude reliable assessment. Of 44 individuals consecutively admitted to the hospital and screened for eligibility, four were excluded prior to enrolment: two declined participation and two could not be included due to severe limb apraxia that prevented them from following pointing instructions required by the assessments. Of the remaining 40 participants who met inclusion criteria, three were discharged from the clinic before completing the second assessment and therefore could not be followed into P2 (see Appendix E in [38]). Participants with at least rudimentary verbal output in either a brief conversation or an informal picture-naming task (5 high-frequency German words, such as “house” and “car”) were assigned to the AAT/ANELT-group. Participants with severe aphasia and/or absent functional verbal expression, e.g., due to apraxia of speech, were assigned to the BIAS-R/Scenario Test group. Three participants whose linguistic data had been included in the analysis by Rubi-Fessen et al. [38] could not be included in the present analysis due to incomplete communicative data sets. The final overall study sample comprised 34 participants (21 men, 13 women; mean age 61 ± 12 years; mean time post-onset 58 ± 34 days) who completed the full study protocol.

All participants, or their legal caregivers where applicable, provided written informed consent prior to enrolment. The study was conducted in accordance with the ethical principles of the 2008 Declaration of Helsinki and received approval from the local institutional review board of RehaNova (April 2019). Table 1 provides an overview of participant characteristics for the slightly reduced sample with data available for the functional communication measures reported on in this paper.

### 2.3. Clinical Assessment

#### 2.3.1. General Disability

The degree of overall functional disability was rated at the beginning of the inpatient stay using the Early Rehabilitation Barthel Index (ERBI) [49] and the Functional Independence Measure (FIM) [50]. ERBI ratings were made by the ward physician, and FIM ratings by nursing staff not involved in the study.

#### 2.3.2. Linguistic Assessment

Depending on the severity of aphasia and verbal-expressive abilities, participants were assessed with one of two severity-specific, standardized German language test batteries: the Aachen Aphasia Test (AAT) [39] for participants with at least rudimentary verbal output (n = 27), or the Bielefeld Aphasia Screening Rehabilitation (BIAS-R) [40] for participants with severe aphasia and/or absent functional verbal expression due to apraxia of speech (n = 10). The rationale for this severity-specific test assignment, the psychometric properties of both instruments, the derivation of spontaneous recovery correction factors for the AAT sample, and the primary (AAT profile level T-score, BIAS-R mean percentage value T-score) and secondary linguistic outcome measures (AAT and BIAS-R subtest scores, AAT spontaneous speech ratings) have been described in detail in our preceding publication [38].

#### 2.3.3. Assessments for Verbal and/or Nonverbal Functional Communication

The central aim of the present study was to examine whether the effects of adjuvant tDCS extended beyond formal linguistic performance to encompass the functional communicative competencies that are more directly relevant to patients’ everyday lives and quality of life. For this reason, assessment instruments measuring verbal and nonverbal communication in ecologically valid, everyday-like situations were administered alongside the linguistic test batteries at all three assessment time points. The use of severity-adequate communicative assessments was a deliberate methodological choice intended to ensure floor-effect-free measurement of functionally relevant communicative change across the full severity range represented in the study sample. Together, these complementary assessment approaches allowed us to investigate both the linguistic and the communicative dimensions of tDCS-associated recovery, and to examine the relationships between changes in these two aspects, which was considered essential for a comprehensive evaluation of the effects of adjuvant tDCS in subacute aphasia.

For the less severely affected group (formerly labelled AAT-group), which was able to communicate verbally, verbal communicative performance was assessed using the Amsterdam–Nijmegen Everyday Language Test (ANELT) [18]. The ANELT presents participants with brief oral descriptions of realistic everyday scenarios—such as calling a doctor’s office—and asks them to respond verbally as they would in the actual situation, approximating a structured role-play format. Versions 1 and 2, with a total of 20 test items (10 items each), were administered, and performance was rated using the A-scale (intelligibility of communicative message), yielding a total raw score, which served as the primary outcome measure for verbal communication. The test specifically evaluates the effectiveness of verbal message transmission in context-bound communicative situations, independent of linguistic accuracy or grammatical correctness. The ANELT has been shown to have high reliability and validity, as well as substantial correlations with the AAT communication rating scale [18]. One PwA was not assessed initially with the ANELT, leaving n = 26 in the less severely affected group (here labelled ANELT-group). For technical reasons, four PwA were only administered Version 1 of the ANELT, leaving n = 22 PwA for all analyses involving Version 2 of the ANELT (see also Table A1).

For the more severely affected group (formerly labelled BIAS-R group), verbal and nonverbal communicative performance was evaluated using the Scenario Test [17]. The Scenario Test was designed specifically for individuals with severe aphasia who rely heavily on multimodal and nonverbal communication strategies. It presents a series of structured everyday communicative scenarios and rates the degree to which a message is successfully conveyed through any communicative means available to the participant, including speaking, gesturing, writing, drawing, or the use of a communication device. The Scenario Test therefore captures communicative success in a genuinely multimodal sense, going well beyond what verbal production measures can reflect, and is particularly suited for individuals with aphasia whose communicative repertoire has shifted substantially toward nonverbal channels. Scored outcomes include the total raw score and the number of successfully transmitted propositions, which served as primary outcome measures for nonverbal communication. Information regarding performance on the Scenario Test was only available for n = 8 PwA (here labelled Scenario-group).

All language and communicative assessments were administered by a single trained examiner (IRF). All verbal responses were digitally recorded, and Scenario Test sessions were additionally recorded on video and audio to allow for the subsequent scoring of nonverbal communicative behaviours—including gesturing, writing and drawing. Transcription and scoring of verbal responses as well as the analyses of nonverbal responses were performed by an experienced research assistant who was blinded to the treatment condition.

### 2.4. tDCS Treatment

Anodal transcranial direct current stimulation was administered using a battery-powered constant-current stimulator (DC-Stimulator mobile, NeuroConn GmbH, Ilmenau, Germany) with a pair of saline-soaked surface sponge electrodes (anode: 5 × 7 cm^2^; cathode: 7 × 10 cm^2^). A constant current of 2 mA was delivered at the onset of the concurrent behavioural therapy session for a duration of 20 min, in accordance with current safety standards for clinical tDCS use [25,27].

As described in detail in Rubi-Fessen et al. [38], stimulation site selection was individualized based on structural CT or MRI imaging, interpreted by two independent investigators (KG and IRF). Three left-hemispheric electrode positions were used, selected according to a hierarchical, lesion-guided protocol: (1) anodal stimulation over the left inferior frontal gyrus (IFG, Broca’s area), (2) anodal stimulation over the left primary motor cortex (M1, position C3), and (3) anodal stimulation over the left frontopolar prefrontal cortex (FPC, position FP1).

In all montages, the reference electrode was placed over the right supraorbital or right frontal region at a minimum distance of 10 cm from the anode. The larger cathode electrode was deliberately chosen to minimize current density beneath the reference electrode and thereby avoid unintended bilateral bipolar stimulation effects [51].

The first combined SLT and tDCS session was conducted jointly by the investigator and the treating SLP to monitor tolerability and familiarize the participant with the procedure. Subsequent sessions were carried out by the responsible SLP. All SLPs at the clinic received structured training in tDCS equipment handling and safety monitoring prior to participation in the study. After each tDCS session, participants rated any discomfort or pain experienced on a visual analogue scale (VAS) ranging from 0 (no discomfort) to 10 (maximum discomfort), and the treating SLP recorded any observed adverse effects such as skin reddening at the electrode site.

### 2.5. Behavioural Therapy

All participants received individualized, multimodal SLT tailored to their specific linguistic impairment profile, severity level, and personal communicative goals. A multimodal therapy framework was applied throughout, targeting the specific language functions impaired in each participant while simultaneously engaging the functional neural networks underlying language processing [52,53]. The specific content of each participant’s therapy was determined collaboratively between the investigator and the treating SLP following the first linguistic and communicative assessment (T1). Where possible, goals were formulated jointly with the participant in accordance with the principle of collaborative goal-setting [54,55]. Where direct participation in goal formulation was not feasible due to severity of impairment, information was gathered from relatives or caregivers regarding the participant’s communicative priorities, personal interests, and daily routines.

In patients without spontaneous verbal expression and severe aphasia, therapy focused on improving oral and written comprehension; verbal recall of relevant and familiar words and formulaic phrases was stimulated within the therapeutic setting; concurrently, alternative communication channels and modalities—such as drawing and gesture—were systematically incorporated into the therapeutic framework, whereby it should be noted that all nonverbal channels were stimulated equally. Wallace et al. [16] had already implemented a similar multimodal therapeutic approach in an acute care setting—an approach that had earlier been tested by Purdy and Van Dyke [14] in patients with chronic aphasia. For less severely affected participants, the emphasis shifted toward deliberate retrieval of words and sentences in verbal and written modalities, as well as more challenging auditory and written language comprehension tasks. Item selection was guided by linguistic criteria including lexical frequency, syntactic complexity, and phonological structure, and was individually adapted to each participant’s profile. The methodology and thematic focus of the behavioural therapy were held constant across P1 and P2 for each participant, with only the level of complexity adjusted to observed changes in language performance, ensuring that any differential improvements between periods could be attributed to the addition of tDCS rather than to changes in therapeutic content.

The content of each session was systematically documented by the treating SLP.

### 2.6. Statistical Analyses

All statistical analyses were conducted using JASP software (version 0.18.3) [56]. For the primary outcome measures, ANELT raw score (A-scale, Version 1 and Version 2) as well as Scenario Test total score and the number of successfully transmitted propositions, repeated-measures ANOVAs with the within-subject factor “time” (3 levels: T1, T2, T3) were carried out. Where the assumption of compound symmetry was violated, degrees of freedom were adjusted according to the Geisser–Greenhouse procedure. Post hoc pairwise comparisons were conducted with Holm correction for multiple testing.

The analyses addressing the core research hypotheses were one-sided *t*-tests applied to post-minus-pretherapy difference scores for each period separately, and to the difference in difference scores (P2 minus P1), which constitutes a direct intraindividual test of the hypothesized superiority of combined SLT and tDCS over SLT alone. For the AAT, in our previous publication, difference scores had been tested also against the expected spontaneous recovery values derived from a historical multicenter spontaneous recovery study for German PwA [57,58]. Since spontaneous recovery information is not available for the ANELT, or the Scenario Test, difference scores for these measures were only tested against zero.

Effect sizes were reported as Cohen’s d with one-sided 95% confidence intervals. To quantify the degree of evidence for or against each hypothesis, Bayes factors (BF_+0_ or BF_0+_, using the default Cauchy prior with r = 0.707) were computed alongside classical significance tests, allowing for a graduated assessment of the strength of evidence, including, importantly, evidence in favour of a null effect [59]. Since the empirical findings so far are that appropriately chosen SLT in post-stroke aphasia is neither harmful nor is the additional, proper application of tDCS in combination with SLT detrimental for the SLT outcome, all intraindividual performance comparisons between post- and pre-treatment periods as well as direct between-performance differences for a SLT-period with additional tDCS against without additional tDCS were carried out as one-sided tests. Two-sided tests would waste statistical power for taking care of a most unlikely outcome, i.e., a detrimental effect of SLT in the population of post-stroke PwA as well as a detrimental add-on effect of tDCS. Correlations between linguistic and communicative outcome measures were computed using Pearson product-moment correlation coefficients.

A sensitivity analysis using G*Power 3.1 [60] indicated that for the ANELT sample (n = 26, resp. 22) the one-sided one-sample *t*-test would be adequately powered to detect effect sizes of d ≥ 0.67, resp. d ≥ 0.74 (α = 0.05, β = 0.95) and d ≥ 0.49, resp. d > 0.55 (β = 0.80); for the smaller Scenario sample (n = 8), the corresponding detectable effect sizes were d ≥ 1.30 (β = 0.95) and d ≥ 1.05 (β = 0.80).

## 3. Results

Because the participants were allotted to separate groups based on the severity of their speech-related symptoms and were therefore assessed with a different combination of diagnostic instruments, both groups (ANELT sample, n = 26 resp. 22; Scenario sample, n = 8) were evaluated separately for their treatment outcomes (see Table A1 and Table A2 for individual performance data). Prior to assessment, the two groups with data for the primary outcome measures were again analyzed for comparability in sociodemographic and basic neurological aspects. (See Appendix Table A3 in Appendix D in Rubi-Fessen et al. [38] for individual patients’ characteristics. PwA 1 did not have an ANELT Version 1 score; PwA 1, 3, 10, 14, and 15 were also lacking an ANELT Version 2 score; and PwA 28 and 29 were without Scenario Test performance data.)

### 3.1. Group Comparability

The gender, age, and years of education distributions did not differ significantly between the ANELT and the Scenario sample, nor did the distribution when leaving out the PwA with missing values for the communication outcome variables (all *p* > 0.10). The Scenario sample had a significantly longer mean duration post-onset (*p* < 0.05 resp. *p* = 0.07 for the n = 22 ANELT subsample) and was in general more severely impaired, as indexed by the ERBI score (*p* < 0.001) and the FIM score (*p* < 0.05). Most PwA in the ANELT sample were in the early subacute phase, in contrast to about half of the PwA in the Scenario sample.

For the Scenario sample, the overall gross aphasia severity grading was based on the BIAS-R average percentage value stanine (SN) score: severe = SN 1–3, moderate = SN 4–5, mild = SN 6–7, and residual = SN 8–9. For the ANELT sample, this overall gradation employed the AAT profile level SN grading with the same four levels. Overall, aphasia severity was exclusively severe in the Scenario sample but covered severe to mild cases in the ANELT sample. As regards fluency of speech output, the Scenario sample contained almost exclusively PwA with non-fluent output, while there were more PwA of the fluent type in the ANELT sample. Stimulation site for the ANELT sample was almost exclusively over the IFG, whereas the sites were more varied for the Scenario sample (IFG, M1, FPG).

### 3.2. Outcome Measures

#### 3.2.1. Comparison Between Assessments at T1, T2, T3

**Figure 1:**
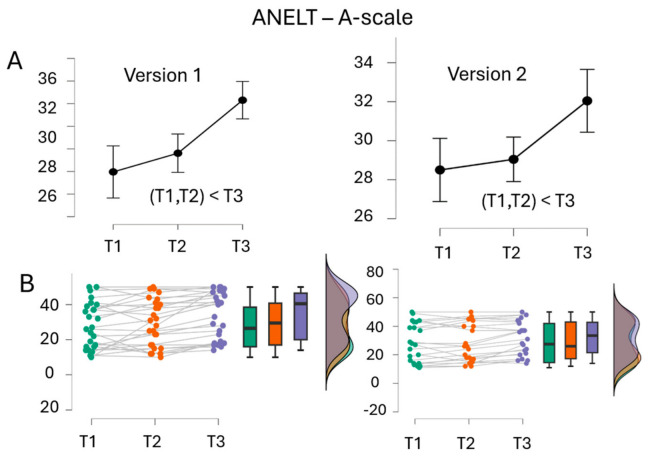
ANELT scale A outcome measures (Version 1: n = 26; Version 2: n = 22). (**A**) Repeated-measures ANOVA cell means profile including within-subject 95%-CI ( for ANELT total scores, Version 1 and Version 2 (max. 50); (**B**) raincloud plots of individual performance courses including boxplots and density estimates.

**Figure 2:**
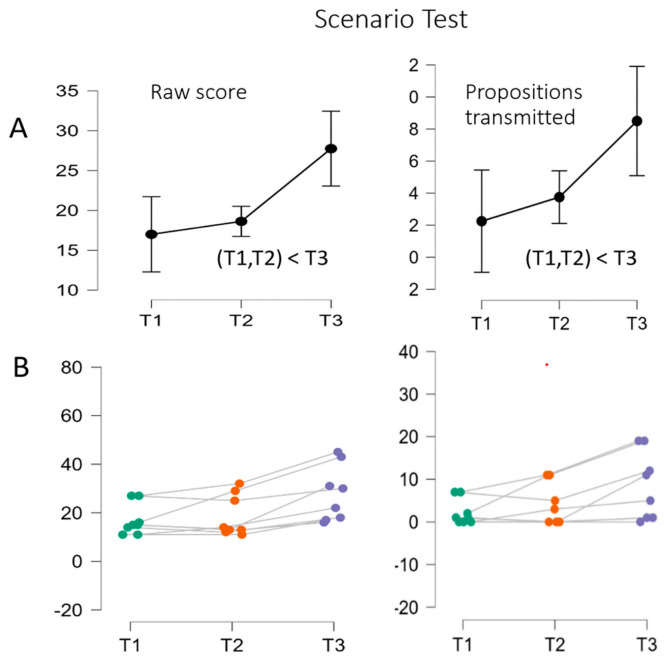
Scenario Test outcome measures (n = 8). (**A**) Repeated-measures ANOVA cell means profile including within-subject 95%-CI () for total performance score (max. 54) and number of propositions transmitted via any communication channel (max. 24); (**B**) individual performance courses for total performance score (max. 54) and number of propositions transmitted via any communication channel (max. 24 prop.).

The three language assessments, taken at pretreatment (T1), post-SLT period 1 (T2), and post-SLT + tDCS treatment period 2 (T3), for the two primary profile level outcome measures yielded a strong overall effect across time with significant improvement in performance for both therapy periods separately (see Table 2 and Figure 1 and Figure 2). As the raincloud plots for the individual courses of performance across therapy show, the improvements in performance look quite homogeneous with only minor crossings of individual courses, if present at all. The statistical findings were qualitatively the same when discarding the one PwA with FPC as the stimulation site.

#### 3.2.2. Comparison Between Post-Minus Pretherapy Difference Score

**Figure 3:**
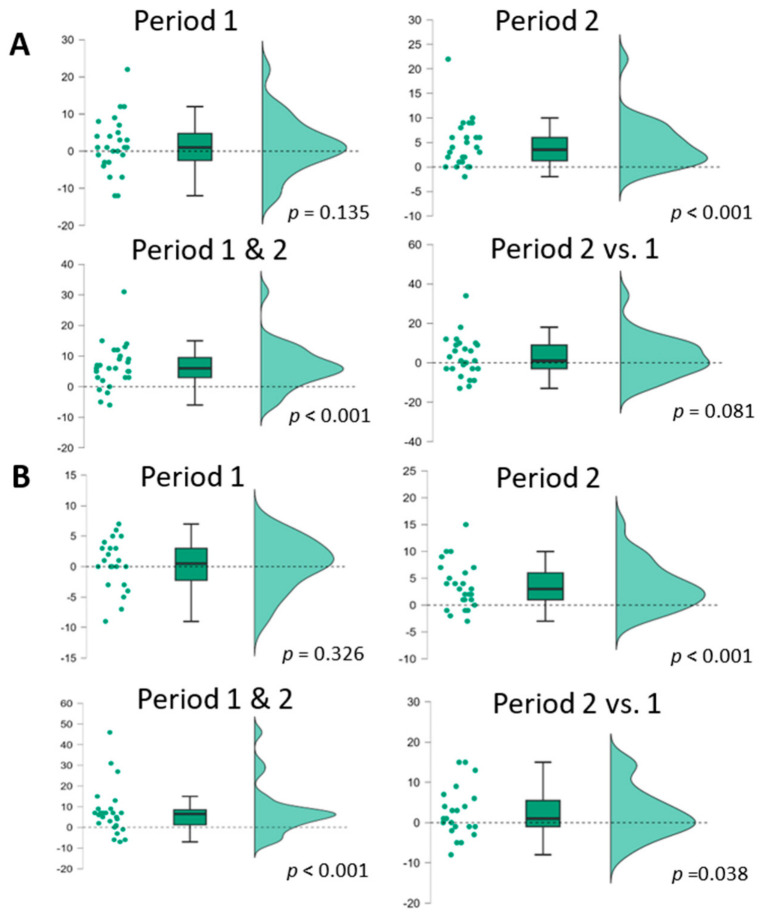
Raincloud plots of ANELT A-scale performance changes across individual period 1 and period 2, both periods 1 and 2 together, and for period 2 compared two period 1. (**A**) Version 1 and (**B**) Version 2.

**Figure 4:**
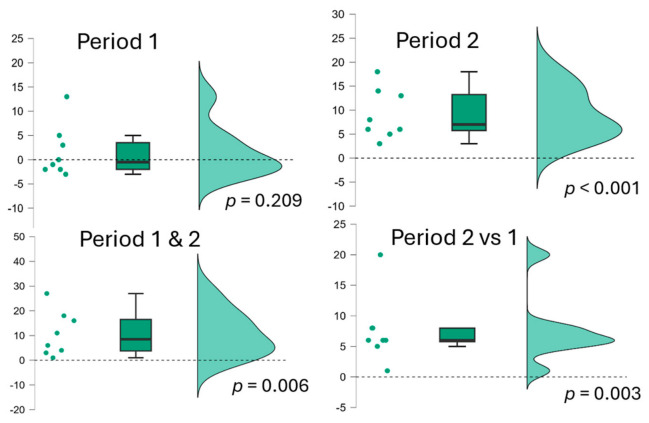
Raincloud plots of Scenario Test performance changes for the total raw score across individual period 1 and period 2, both periods 1 and 2 together and for period 2 compared to period 1.

More relevant for the specific research questions of this study are analyses of the post-minus pretherapy period difference scores tested one-sided against zero (no effect across the therapy phase) separately with a *t*-test for both periods and the direct comparison of the difference score for P2 minus the difference score for P1, tested one-sided against zero (no difference in effect of therapy for SLT + tDCS vs. SLT alone) with a *t*-test as well (see Table 3 and Figure 3 and Figure 4).

For the AAT A-scale in therapy P1, there is no significant improvement for both test versions, with the Bayes factor indicating moderate evidence for the null hypothesis of no change.

The situation is different for P2 with added tDCS stimulation. The average difference score is significantly larger than zero, with a large Cohen’s d effect size estimate and very strong evidence that the alternative hypothesis of positive change is true (see Table 3A). Cohen’s d is above the limit of 0.67 resp. 0.74 from the sensitivity analysis. As the raincloud plot in Figure 3A,B nicely reveals, the great majority of PwA in the ANELT group had a positive change in performance. Moreover, the improvement after the combined therapy was numerically higher than the improvement after SLT alone (see Figure 3A and Table 3A), but failed to reach significance, in particular for Version 1 of the test; for Version 2 the differential effect was significant only at the 5%-level and not when correcting for multiple testing. Furthermore, there was a large significant improvement in performance across the whole treatment phase of four weeks with strong evidence (see Figure 3A,B and Table 3A), the effect estimate again being around or beyond the threshold from the sensitivity analysis.

For the Scenario group and both dependent variables, total quantitative raw score and number of propositions successfully transmitted via any communicative channel, a numerically qualitatively similar pattern of effects was present (see Figure 4 only for the raw score and Table 3B) for all one-sided *t*-tests against the null hypothesis of no change although not all tests revealed significant improvement, possibly also due to the small sample size. Here, as for the ANELT in P1, no significant improvement was found. The differential superiority effect of the combined treatment was only present for the total quantitative score with strong evidence.

### 3.3. Additional Analyses

#### 3.3.1. Responders to Treatment

Another perspective on treatment success is looking for the number of “responders”, i.e., PwA whose individual difference score is above the critical difference for a reliable improvement in performance (also called reliable change or minimal detectable change MDC). For the ANELT, this critical difference is seven raw scores for both versions [18]. In the first therapy period, 6/26 = 23.1% of the PwA improved significantly for Version 1 and 2/22 = 9% for Version 2; this proportion was 7/26 = 26.9% for the combined therapy period P2 (resp. 5/22 = 22.7% for Version 2). In the Scenario group, there was only one responder in period 1 (1/8 = 12.5%) with a quantitative score increase of at least 7 points (see Table 4), reported as the critical difference for the German version of the test [17]. This PwA again and another three individuals were responders for the combined therapy period 2 (4/8 = 50%).

#### 3.3.2. Inter-Individual Differences in the Scenario Test

The individual profiles of communication channels employed by the PwA in the different scenarios at all three assessments, as reported in Table 4, show a considerable variability in and preference for channels used with no clear-cut relation to the quantitative score level of performance, also listed in Table 4. Next to speaking, drawing was the most frequent nonverbal mode of communication, whereas writing and gesturing were utilized only very rarely, as well as combining channels.

The trajectories of total raw scores across therapy phases show a broadly consistent pattern of improvement across participants. Following pperiod 1, only PwA 7 exceeded the critical difference threshold of 7 raw score points; PwA 6 showed a numerical improvement that remained below this threshold. All remaining participants showed only minimal positive or negative score changes, with individual variation not exceeding 3 points. Following Period 2, all eight participants improved numerically relative to T2. Four out of eight participants exceeded the critical difference threshold of 7 points; two further participants narrowly fell below this threshold with gains of 6 points each.

With respect to the number of propositions successfully conveyed and the communicative modalities employed, the individual profiles showed considerable heterogeneity across the full therapy period. The total number of propositions conveyed increased continuously across all time points in five of the eight participants; the remaining three showed a non-continuous improvement pattern, including two participants whose scores temporarily declined at T2 before recovering at T3. The magnitude of improvement varied substantially both across individuals and across therapy phases. Three participants conveyed a maximum of one proposition even at T3, while all remaining participants conveyed at least five propositions, with the highest individual score reaching 19 out of 24 possible propositions. In all but one participant, the increase in successfully conveyed propositions was greater following P2 than following P1, with the additional gain attributable to P2 ranging from 1 to 11 propositions.

The communicative modalities used for successful proposition transmission also varied substantially across participants and assessment time points. At T1, speech was the preferred modality, with writing, drawing, gesture, and combined modality use additionally represented across participants. At T2, speech remained the primary modality; drawing use increased relative to T1, while gesture use decreased. At T3, following period 2, six out of eight participants conveyed at least one proposition verbally; four out of eight used drawing, with three of these employing it as their primary communicative modality. Individual propositions were also conveyed via writing and combined modalities. Notably, gesture was not employed as a successful communicative modality by any PwA at T3.

### 3.4. Correlational Analyses

Correlational findings will only be reported in detail for the ANELT sample, because the Scenario sample is too small for detecting reliable correlational associations, in particular. Overall, for the BIAS-R group, correlations between BIAS-R (MPV) and Scenario Test measures showed a systematic decrease from T1 (r = 0.78) via T2 (r = 0.49) to T3 (r = 0.34).

**Figure 5:**
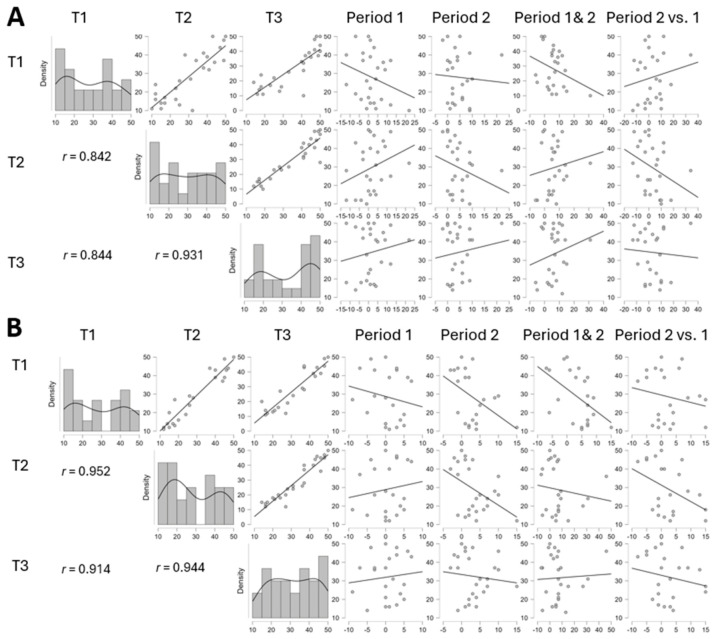
Scatterplots and correlation coefficients among levels of performance for the ANELT total raw score Version 1 in (**A**) and Version 2 in (**B**) for all three pairs of assessments; for relations between performance levels and changes in performance across therapy periods, only scatterplots are shown without reporting the non-significant correlation coefficients after adjustment for multiple testing.

Figure 5 shows density plots in the diagonal, scatter plots and correlation coefficients among the ANELT total scores for both versions separately for the three individual assessments and their change scores. There is a very high correlation among assessments of between 0.84 and 0.95, in particular for Version 2 (*p* < 0.001 for all three correlations between pairs of time points for both ANELT Versions A and B). Interestingly, the initial performance level is not strongly correlated with the change in performance for the first period, the second period, the whole therapy phase, or the differential change in P2 versus P1 (all correlations below 0.50 or much less; only scatterplots but no actual values of non-significant correlations—adjusted for multiple testing—shown in Figure 5).

Age was not related to any of the assessments or to the change scores. Duration was moderately negatively correlated with the initial period change score (r = −0.54, *p* < 0.01) and somewhat less so with the change score across the whole treatment phase (r = −0.41, *p* < 0.05) and the differential change score (r = −0.2, *p* < 0.01), thus favouring PwA with a somewhat longer duration in the early phase post-onset.

A comparison of correlation and intraclass correlation (ICC) between the three pairs of assessments for the ANELT total score—as shown in Table 5—demonstrates that the correlation coefficient may not be the most adequate measure of retest reliability or stability in performance when there is a varying degree of change in the level of performance across assessment occasions, in particular for period 2 and both periods taken together. A one-factor repeated-measures ANOVA with two time points was carried out first in JASP to obtain the mean squares for factor time with two levels, for (random effects) factor PwA with n = 26 resp. n = 22, and the residual mean squares, in which interaction between time and PwA (differences in steepness of slope) and additional random error are confounded. Also, both test versions were highly correlated for each of the three assessments.

#### Correlation Between ANELT Communication Scores and AAT Scores

There is a quite consistent pattern of correlations between AAT performances and ANELT total scores for both versions across all three assessments (see Table 6). For the spontaneous speech rating scales, there is a tendency for increasing correlations from T1 to T3. The numerically highest correlations are for communicative behaviour (COM), syntax (SYN), and semantic structure (SEM). Among the AAT subtest performance variables, there is the highest correlation with the profile level (reliability weighted average among subtest T-scores), subtest Confrontation Naming, and increasingly over the three time points with subtest Comprehension. Interestingly, performance on the AAT does not show substantial correlations with changes in ANELT performance scores for both versions.

## 4. Discussion

The present observational study provides the first evidence of an additive effect of anodal tDCS on both verbal and nonverbal functional communication outcomes of aphasia therapy in individuals with subacute aphasia. Among less severely affected participants, communicative performance improved significantly more during tDCS-supported P2 compared to P1 on Version 2 (numerical tendency only for Version 1) of the ANELT A-scale; among those with severe aphasia and markedly impaired verbal expression, adjuvant tDCS resulted in significantly greater gains in Scenario Test total score and number of propositions successfully conveyed in P2. The additive effect of tDCS on formal linguistic performance, as measured by the AAT and the BIAS-R, has been reported in detail in our preceding publication, Rubi-Fessen et al. [38]. Whilst spontaneous remission cannot be excluded as a contributing factor to the observed gains, it is worth noting that improvements were consistently greater during P2 than during P1—despite the fact that the influence of spontaneous remission would be expected to diminish rather than increase over time. This asymmetry therefore supports the interpretation that the superior gains observed in P2 reflect a genuine treatment-related add-on effect rather than a continuation of natural recovery.

The feasibility of implementing an individually tailored stimulation protocol within the routine of an early rehabilitation clinic, the good tolerability of the stimulation, and the absence of adverse side effects, as well as the methodological limitations inherent to this approach, have likewise been discussed in Rubi-Fessen et al. [38]. Given the heterogeneity of lesion patterns and the wide range of severity and clinical presentation in the early subacute phase, a uniform stimulation site and fully standardized assessment protocol are unlikely to be appropriate for this patient population. Similarly, a crossover design—as frequently employed in studies of chronic aphasia—is not suitable in the early subacute stage, both because of the confounding influence of spontaneous remission and because an adequate washout period between active and sham stimulation (e.g., two months, as applied by Kim et al. [43] cannot be maintained within the timeframe of an inpatient rehabilitation stay.

### 4.1. Verbal Communication: Improvements on the ANELT A-Scale

On both versions of the ANELT, performance showed only a numerical, non-significant improvement on average during P1, in contrast to the significant gains observed during tDCS-supported P2. A direct comparison of P1 and P2 revealed a numerical trend toward superiority of the combined treatment for Version 1 and a significant add-on effect for Version 2. This pattern is noteworthy in light of the well-documented challenge that improvements in formal linguistic measures do not automatically translate into functional communicative gains [12,19,20]. Indeed, already in P1, significant improvements were observed in the verbally expressive subtests of the AAT—including Repetition and Naming—at least prior to correction for spontaneous remission; yet at the same time, ANELT performance showed only marginal numerical change. Given that both AAT naming tasks and the successful completion of ANELT scenarios require effective word retrieval, the additional anodal stimulation of the left IFG—administered to all participants except one in the ANELT Version 1 group and 21/22 in the Version 2 group—may have provided the additional excitatory drive necessary for this retrieval capacity to generalize to functional communicative contexts in P2. It is worth noting that intensive impairment-based behavioural aphasia therapy without adjuvant brain stimulation has itself been shown to be followed by improvements in functional verbal communication [22,61,62]; the present findings suggest that tDCS may further facilitate this generalization, at least in the subacute phase.

Our results stand in contrast to those of Spielmann et al. [36], who applied tDCS over the left IFG in a multicenter RCT with individuals with subacute aphasia and found no significant effect on either naming or ANELT performance. Two methodological differences may account for this discrepancy. First, Spielmann et al. used a lower stimulation intensity of 1 mA compared to the 2 mA applied in the present study. Second, and potentially more importantly, Spielmann et al. did not use structural imaging to check that viable, reactivatable tissue was present beneath the stimulation electrode in the left IFG—a prerequisite that was systematically controlled for in our protocol. Stimulating over necrotic or severely damaged tissue is unlikely to engage the residual language network effectively, and the absence of this control may have contributed to the null result.

The only other study to systematically examine tDCS effects on both linguistic and communicative outcomes in subacute aphasia is Stockbridge et al. [37], who reported significant improvement in verbal functional communication—assessed via a picture description task—in the active tDCS group, in the absence of a significant naming effect. This dissociation between task-based linguistic and connected-speech communicative outcomes parallels our own finding of a more robust communicative than linguistic add-on effect in P2 and supports the view that discourse-level communicative performance may be more sensitive to tDCS-induced network effects than single-word measures.

A growing number of studies in chronic aphasia have likewise documented positive effects of tDCS on verbal communicative outcomes [42,43,44]. It should be noted, however, that the functional communication measures employed in these studies were not performance-based task assessments, but rather subjective proxy ratings completed by caregivers or clinicians. The present study is, to our knowledge, the first to demonstrate a tDCS-associated gain in verbal functional communication in subacute aphasia using a standardized, performance-based, task-specific instrument, the ANELT, that directly captures communicative effectiveness in ecologically valid everyday scenarios.

### 4.2. Relationships Between Linguistic and Communicative Measures: AAT and ANELT

A consistent pattern of moderately to highly significant correlations was found between AAT variables—including profile level, spontaneous speech ratings, and subtest scores—and the ANELT A-scale total score for both versions across all three assessment time points, with the exception of the articulation and phonology subscales of the AAT spontaneous speech analysis. The strongest correlations were observed between the ANELT A-scale and the AAT communicative behaviour scale—part of the spontaneous speech analysis—and the naming subtest, which is consistent with the overlapping task demands of these measures. The robust association between the AAT communicative behaviour scale and the ANELT A-scale had already been documented by Blomert et al. [18]. The weaker but nonetheless significant correlations observed with other expressive and receptive AAT subtests may reflect a more general activation of the language network, driven jointly by the still-active process of spontaneous remission and the network-level excitatory effect of anodal tDCS over the left IFG—a region that functions as a critical hub within the distributed language network. Further studies have reported a positive effect of non-invasive brain stimulation on the relationship/association between linguistic and communicative improvements. Kim et al. [43] found, following cerebellar tDCS combined with naming training in n = 24 individuals with chronic aphasia, a correlation between practiced items and the (ASHA-FACS)-scale [45]. They, too, explain the effect of tDCS through additional activation—in this case, of the cerebro-cerebellar loop—which strengthens lexical–semantic representations and their connections, leading to improved naming.

Of particular interest are the trajectories of individual correlations over time. For communicative behaviour and naming, the correlation with the ANELT A-scale remained stable or increased across assessment time points; correlations between the ANELT and skills underlying successful verbal communication in context—including semantics, syntax, and language comprehension—which were initially weaker, showed an increasing trend over the course of therapy.

The potentially differential contribution of linguistic domains to functional communicative performance, as captured by the ANELT, is further supported by the findings of Doesborgh et al. [63], who investigated the impact of phonological and semantic deficits on ANELT A-scale outcomes and identified semantic performance as the only strong predictor of ANELT scores—a finding consistent with the absence of significant correlations between the ANELT and the articulation and phonology subscales over time observed in the present study. This also allows for the identification of clinically relevant therapeutic implications, with a preference for lexical–semantic therapy over phonologically oriented therapy.

### 4.3. Nonverbal Communication: Improvements in the Scenario Test

For the Scenario test, no significant changes were observed during P1, in contrast to the significant increases observed during P2. A direct comparison of P1 and P2 revealed a significant add-on effect for the tDCS-supported P2. The analysis of the number of propositions successfully conveyed, regardless of modality, showed only an average numerical but not statistically significant increase in P1, but a statistically significant increase for P2 with additional tDCS, as well as a marginally significant superiority of the second tDCS-supported therapy phase.

These findings are notable in the context of the broader assessment literature. Despite the fact that the Scenario Test is one of only four standardized instruments included in the proposed Core Outcome Set for aphasia trials within the international ROMA-2 project for the standardization of outcome measures [64], and despite being identified in a critical review of functional communication measures as the most suitable instrument for the quantitative assessment of multimodal communicative abilities in aphasia [20], to our knowledge no published aphasia therapy study has employed it as an outcome measure—with the exception of the original validation studies of the Dutch and UK versions [65,66], and a study by Gauch et al. [67] who employed the Scenario Test to compare modality differences between tele-practice and face-to-face sessions in individuals with dementia-related speech disorders rather than post-stroke aphasia.

**Figure 6:**
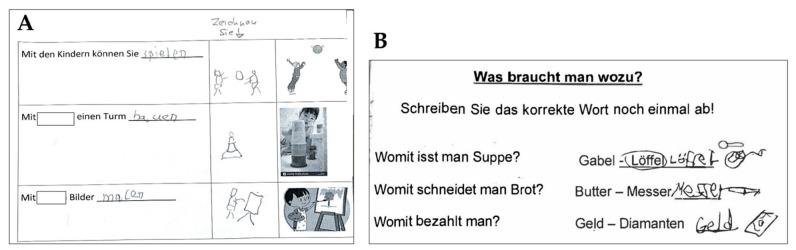
Examples from individual multimodal therapy comprising reading/auditory comprehension, drawing, writing and gesturing: (**A**) Related to activities with the PwA’s children (names covered)—“playing, “building a tower”, “painting pictures”—and (**B**) concerning to functional relationships with everyday objects—“what do you use to eat soup? → spoon”.

Targeted training in specific nonverbal communication strategies—such as gesture therapy, communicative drawing, or technology-based AAC—has been shown to produce gains in the trained modality in chronic aphasia [68,69,70,71]. However, as discussed in systematic reviews of gesture treatment, generalization of these modality-specific gains to functional everyday communication has rarely been demonstrated [72,73]. Crucially, in the present study, no specific training of an isolated verbal or nonverbal modality was conducted during either therapy period. Rather, both verbal modalities—speech and writing—and nonverbal communicative channels—gesture and drawing—were systematically stimulated as part of the individualized multimodal SLT approach applied throughout P1 and P2 (see Figure 6 for examples).

In contrast to linguistic performance, which showed significant group-level improvement during P1 even in the Scenario group (see Table A2), no significant gains were observed in mean Scenario Test scores during the same period. However, one of the eight participants achieved both a significant increase in raw score (responder) and a substantial increase in the number of propositions conveyed via the drawing modality during P1 alone. During P2 with adjuvant tDCS, four out of eight participants exceeded the critical difference responder threshold of at least 7 raw score points; two further participants narrowly fell below this threshold. The overall trajectory across the full four-week treatment phase—from a mean raw score of 17.00 at T1 to 27.75 at T3—is broadly comparable to the trajectory observed in the nonverbal subgroup (n = 43) of the Dutch validation sample, which increased from a mean of 22 to 35.3 raw score points, albeit over a six-month period [65]. Within our sample, the steepest improvement occurred during P2, with mean raw scores rising from 18.63 at T2 to 27.75 at T3. It should be noted, however, that van der Meulen et al. [65] do not report the extent or type of therapy received by participants in the Dutch validation sample during the six-month interval between assessments, which limits the interpretability of this comparison.

A particularly informative finding from the individual response data is the emergence of distinct communicative profiles across participants in the Scenario Test, reflecting different adaptive strategies for transmitting information. Three broadly distinguishable response patterns were observed. The first, which may be termed the verbal profile, characterized participants (Table 4: PwA 31 and 33) who were already able to retrieve some contextually appropriate single words at T1, maintained or slightly extended this strategy at T2, and showed a marked increase in the number of verbal propositions successfully conveyed at T3. The second, the drawing profile, characterized participants (PwA 34, 35 and partially 36) who began using drawing as a communicative strategy for the first time at T2 and showed a substantial increase in the number of propositions conveyed through drawing following P2.

While both PwA 34 and PwA 35 used drawing as their primary communicative modality, their profiles differed in important respects. Both presented with severe apraxia of speech in addition to aphasia, which further impeded verbal word retrieval. PwA 35, however, was able to employ drawing strategically to facilitate verbal recall to some degree, whereas PwA 36 relied on drawing purely as a compensatory strategy. This distinction is consistent with findings by Farias et al. [74], who propose that drawing may facilitate naming by activating a widely distributed semantic network. In individuals with aphasia following left-hemispheric stroke, it is hypothesized that drawing may contribute to naming by engaging residual semantic representations in the right hemisphere and intact perilesional areas of the left hemisphere. Given the extensive frontal lesion in PwA 35, it is plausible that no residual left-hemispheric areas capable of supporting verbal facilitation remained, rendering drawing a purely compensatory rather than a facilitative channel. The third, the comprehension-based profile, characterized participants (PwA 30, 32, and 37) who were able to convey at most one proposition through speech or gesture across all time points but achieved meaningful improvements in their Scenario Test total score—in some cases considerably—by responding more accurately to the yes/no questions embedded in the scenarios.

One notable finding across the sample was the reduced use of gesture over the course of therapy. Although gesture training has been shown to be effective for practiced items in chronic aphasia [68,72], and despite the fact that limb apraxia had been excluded as an eligibility criterion and gesture was consistently stimulated as part of the multimodal SLT across both therapy periods, this modality was not employed as a successful communicative channel in the Scenario Test beyond T1. The reasons for this remain unclear. One possible explanation relates to the nature of the Scenario Test itself, which presents novel, contextually specific communicative situations; unlike trained gesture tasks, these scenarios may not have provided sufficient retrieval support for gestural expressions that had not been practiced in an equivalent format during therapy.

The emergence and flexibility of these profiles may have been facilitated by the consistent multimodal therapeutic approach and the systematic inclusion of language comprehension targets for these severely affected participants—both of which, however, were already in place during P1. A potentially more parsimonious explanation may lie in a form of reduced cognitive load resulting from the increased excitatory activation of residual left-hemispheric language networks induced by tDCS. The flexible use of alternative communicative channels requires a set of executive capacities: the ability to recognize that an initial communicative attempt has failed, to inhibit the predominant verbal response tendency, to switch to an alternative modality, and to initiate and sustain a different communicative strategy [16]. Since inhibitory control and cognitive switching rely on executive functions that are frequently impaired in persons with aphasia [75,76], the additional excitatory input provided by tDCS may have partially relieved these executive demands, thereby facilitating greater flexibility in modality selection and communicative strategy use. In the case of PwA 35—who received stimulation over the left frontopolar cortex—an initial shift in communicative modality preference may additionally have been facilitated by the activation of domain-general frontal networks, which have been shown to contribute to cognitive flexibility and the reorganization of language-adjacent processes following stroke [77,78,79].

### 4.4. Relationships Between Linguistic and Communicative Measures: BIAS-R and Scenario Test

Improvements in linguistic test performance following aphasia therapy do not automatically translate into improvements in verbal functional communication, and even less so into gains in nonverbal communicative competence. Correspondingly, strong correlations between change scores in linguistic and communicative variables would not necessarily be expected. As noted earlier, the Scenario group’s sample size of n = 8 is too small to establish reliable correlations; therefore, our results should be viewed with that caveat in mind. The pattern of correlations between BIAS-R variables and Scenario Test outcomes showed a systematic tendency towards a decreasing magnitude from T1 to T3. This gradual decoupling of formal linguistic performance and functional communicative competence as rehabilitation proceeded might suggest that the two levels of recovery followed partially divergent trajectories. To our knowledge, no prior therapy study has explicitly and systematically investigated and compared the parallel development of linguistic skills and multimodal functional communication in aphasia. The most directly relevant contribution is that of Meier et al. [61], who investigated the relationship between therapy-induced changes in cognitive–linguistic skills and changes in functional communication abilities. Their findings revealed that cognitive–linguistic abilities and functional communicative performance were closely and positively associated at a single time point—consistent with our own T1 correlations—but that this relationship was rarely preserved in pre-to-post-treatment change scores. Meier et al. therefore concluded that although cognitive–linguistic abilities and functional communication are both responsive to the same therapeutic interventions, they constitute connected but nonetheless distinct constructs.

### 4.5. Limitations

The principal methodological limitations of the study design—including the absence of a crossover design, which is commonly employed in tDCS studies of chronic aphasia but cannot be implemented in the subacute phase due to the confounding influence of spontaneous remission—have been discussed in detail in our preceding publication [38]. The most methodologically rigorous approach for confirming the present findings would be a larger-scale multicentre randomized controlled trial, which is currently in the planning stages based on the results obtained to date.

The present findings should be interpreted in light of the small sample sizes, in particular for the BIAS-R and Scenario Test group, which mainly concerns the generalizability of the Scenario results. A further limitation concerns the absence of patient-reported outcome measures (PROMs) or proxy-based measures of quality of life and communicative participation. This gap is partly attributable to the characteristics of the subacute phase itself: no standardized, psychometrically validated instruments are currently available in German that are suited for routine clinical use in this phase. The Stroke and Aphasia Quality of Life Scale—39 generic version (SAQOL-39g), which has recently been published in a validated German adaptation [80] and is widely used in aphasia research, has been validated for the chronic phase only; moreover, several of its items are not well suited to an inpatient rehabilitation context. Similarly, the Communicative Effectiveness Index (CETI) [46], one of the most frequently employed proxy instruments in aphasia research, has not been validated for use in the subacute stage.

## 5. Conclusions

To the best of our knowledge, the present study provides the first evidence of an additive effect of anodal tDCS on therapy-induced improvements in both verbal and nonverbal functional communication in individuals with subacute aphasia. Beyond demonstrating this add-on effect, the study revealed distinct temporal patterns in the relationship between linguistic and communicative outcomes: although the precise parameters for optimal tDCS stimulation protocols in the subacute phase have not yet been definitively established and require further investigation, the present results support the view that individually tailored anodal tDCS is a feasible, well-tolerated, and clinically promising adjuvant tool for enhancing the communicative outcomes of behavioural aphasia therapy in early rehabilitation.

Beyond the specific contribution of tDCS, the present study highlights a broader clinical imperative: the systematic assessment of nonverbal communication—particularly in individuals with severe aphasia—should be considered an integral component of aphasia diagnostics rather than an optional supplement. Standardized, performance-based instruments that capture both verbal and nonverbal functional communication, such as the Scenario Test, provide clinically essential information for therapy planning, the identification of functional communicative resources, and the selection of promising compensatory strategies. Despite the moderate sample size and the observational design of the present study, we confidently conclude that incorporating such assessments routinely, and particularly in the early stages of aphasia rehabilitation, can lay the foundation for fully recognizing and developing the communicative capacities of people with aphasia—thereby supporting their quality of life and meaningful participation in social and everyday life.

## Figures and Tables

**Table 1:** Patient characteristics.

Group		AAT/ANELT (n = 26)	BIAS-R/Scenario Test (n = 8)
Etiology (stroke)	IS	22	6
	IHS	4	2
Stimulation site (left)	IFG	25	3
	M1		3
	FPC	1	2
Sex	Male	18	3
	Female	8	5
Age (years)	M (SD)	62.38 (12.02)	55.26 (8.81)
	Median (range)	63.5 (36–83)	55 (39–66)
Education (years)	M (SD)	14.31 (2.78)	14.1 (3.00)
	Median (range)	16 (10–18)	14.5 (10–17)
Duration (days)	M (SD)	50.73 (27.62)	80.0 (42.81)
	Median (range)	46.5 (15–107)	68 (33–144)
Aphasia type ^1^	Global	5	
	Wernicke	10	
	Broca	6	
	Anomic	5	
	Subacute aphasia		8
Aphasia severity ^2,3^	Severe	9	8
	Moderate	10	
	Mild	6	
	Residual	1	
Severity ERBI ^4^ (−325 to +105)	M (SD)	+18.1 (32.25)	−53.0 (47.20)
	Median (range)	+25 (−65 to +60)	−62.5 (−125 to +40)
Severity FIM ^5^ (18–126)	M (SD)	74.4 (19.99)	55.1 (15.53)
	Median (range)	80.5 (29–107)	54.5 (38–90)

Note: Table 1 reflects the participants’ assignment to the severity-specific assessment structure. IS = Ischemic; IHS = Intracerebral hemorrhagic; IFG = Inferior frontal gyrus; M1 = Motor cortex; FPC = Frontopolar cortex; F = Female; M = Male; ^1^ Aphasia diagnosis according to Aachen Aphasia Test (AAT)/BIAS-R does not classify into aphasia types (subacute aphasia). ^2^ Severity according to AAT profile level value (T-score); severe <46, moderate 46–54.9, mild 55–62.9, residual ≥63. ^3^ Severity according to BIAS-R percentile rank (PR); severe <23, moderate 24–59, mild 62–88, residual ≥89. ^4^ Early Rehabilitation Barthel Index (ERBI), at beginning of rehabilitation stay. ^5^ Functional Independence Measure (FIM), at beginning of rehabilitation stay.

**Table 2:** Descriptive information (arithmetic mean (M), standard deviation (SD)) and inferential statistical analysis of performances across the therapy period. A: ANELT total raw score for both versions; B: Scenario Test total score and number of propositions transmitted.

**A**	**ANELT**	**T1**	**T2**	**T3**	**ANOVA Factor Time**			**Post Hoc** **Compar. ^§^**
	**(n = 26 resp. 22)**	**M (SD)**	**M (SD)**	**M (SD)**	**F(2,50) ^#^**	***p***	**η^2^_p_**	
	Total score, Version 1 (10–50)	27.96 (13.25)	29.62 (13.35)	34.31 (13.11)	12.60	<0.001	0.34	(T1, T2) < T3
	Total score, Version 2 (10–50)	28.50 (13.68)	29.05 (13.46)	32.05 (11.95)	7.24	0.002	0.26	(T1, T2) < T3
**B**	**Scenario Test**	**T1**	**T2**	**T3**	**ANOVA Factor Time**			**Post Hoc** **Compar. ^§^**
	**(n = 8)**	**M (SD)**	**M (SD)**	**M (SD)**	**F(2,14) ^#^**	***p***	**η^2^_p_**	
	Total score (0–54)	17.00 (6.44)	18.63 (8.57)	27.75 (11.51)	11.77	<0.001	0.63	(T1, T2) < T3
	Propositions transm. (max 24)	2.25 (3.01)	3.75 (4.83)	8.50 (7.89)	7.29	0.007	0.49	(T1, T2) < T3

^#^ Adjustment of df in case of lacking compound symmetry according to Geisser and Greenhouse. ^§^ Pairwise comparisons and adjustment of *p*-values for the sequentially rejective multiple test procedure for a family of three tests, overall type-I error level = 0.025 for each test variable under A as well as under B; time points included in brackets under the post hoc comparisons column do not show significant differences in performance.

**Table 3:** Analysis of difference scores between assessments: One-sided *t*-tests for improvement against zero and differentially stronger improvement for period 2 compared to period 1, as well as effect size estimates (Cohen’s d with 95% one-sided confidence interval) and Bayes factor (BF) for the degree of evidence for improvement (BF_+0_) or for lack of improvement (BF_0+_). A: ANELT total score Version 1 and Version 2; B: Scenario total score and propositions transmitted.

**A**	**Period 1**			**Period 2**			**Period 2 − 1**			**Period 1 + 2**		
**ANELT**	t(25) * **(*p*)**	**Cohen d** **[95%-CI]**	**Bayes** **Factor**	t(25) * **(*p*)**	**Cohen d** **[95%-CI]**	**Bayes** **Factor**	t(25) * **(*p*)**	**Cohen d** **[95%-CI]**	**Bayes** **Factor**	t(25) * **(*p*)**	**Cohen d** **[95%-CI]**	**Bayes** **Factor**
**Version 1** (n = 26)	1.13 (0.14)	0.22 [−0.11–∞]	BF_0+_ 2.70	**4.74** (<0.001)	0.93 [0.54–∞]	BF_+0_ 352	1.44 (0.08)	0.28 [−0.05–∞]	BF_0+_ 1.92	**4.67** (<0.001)	0.90 [0.51–∞]	BF_+0_ 324
**ANELT Version 2** (n = 22)	0.46 (0.33)	0.10 [−0.26–∞]	BF_0+_ 4.01	**4.05** (<0.001)	0.81 [0.42–∞]	BF_+0_ 69	1.87 (0.038)	0.40 [0.03–∞]	BF_+0_ 0.97	**3.26** (<0.001)	0.64 [0.28–∞]	BF_+0_ 12
**B**	**Period 1**			**Period 2**			**Period 2 − 1**			**Period 1 + 2**		
**Scenario Test**	t(7) * **(*p*)**	**Cohen d** **[95%-CI]**	**Bayes** **Factor**	t(7) * **(*p*)**	**Cohen d** **[95%-CI]**	**Bayes** **Factor**	t(7) * **(*p*)**	**Cohen d** **[95%-CI]**	**Bayes** **Factor**	t(7) * **(*p*)**	**Cohen d** **[95%-CI]**	**Bayes** **Factor**
Total score	0.86 (0.21)	0.30 [−0.30–∞]	BF_0+_ 1.33	**4.92** (<0.001)	1.74 [0.56–∞]	BF_+0_ 53	**3.86** (0.003)	1.36 [0.50–∞]	BF_0+_ 18.8	**3.34** (0.006)	1.19 [0.39–∞]	BF_+0_ 11.3
Propositions transm.	1.30 (0.12)	0.46 [−0.17–∞]	BF_0+_ 1.06	**3.20** (0.008)	1.13 [0.34–∞]	BF_+0_ 9.34	1.91 (0.049)	0.68 [0.01–∞]	BF_+0_ 2.18	**2.88** (0.012)	1.02 [0.26–∞]	BF_+0_ 6.19

* resp. *t*(21) for ANELT Version 2; in bold font: significant also after Holm correction for family of two tests (ANELT as well as Scenario Test alpha = 0.025 each).

**Table 4:** The communication channels successfully used, and the total quantitative score attained by individual PwA in the Scenario Test group in all three assessments (Sp: Speaking; Wr: Writing; Dr: Drawing; Ge: Gesturing; Co: Combining channels).

	T1						T2						T3					
PwA ^1^	Sp	Wr	Dr	Ge	Co	Score	Sp	Wr	Dr	Ge	Co	Score	Sp	Wr	Dr	Ge	Co	Score
3 (30)				1		15						13						16
4 (31)	5		2			22	5					25	9		1		2	30
5 (32)						11						11	1					17
6 (33)	6			1		27	9	1		1		32	17	2				45
7 (34)	1	1				16	1		9		1	29	4		14		1	43
8 (35)				1		15						13	1		10			31
9 (36)						11			3			14			5			22
10 (37)						14						12	1					18

^1^ No data available for the first two patients in the Scenario Test group; the numbers in brackets correspond to the overall numbering of PwA in Rubi-Fessen et al. [38].

**Table 5:** Intraclass correlations (ICC) for consistency and stability (agreement) of ANELT performance over the therapy periods.

ANELT	Version 1			Version 2			Version 1 × Version 2		
Period	1	2	1&2	1	2	1&2	1	2	1&2
MS (time ^1^) ^#^	35.558	286.231	523.558	3.273	162.000	138.273	10.023	10.580	19.692
MS (PwA) ^#^	325.887	337.837	320.422	359.273	289.847	314.511	345.451	342.370	313.329
MS (residual) ^#^	27.918	12.151	27.038	8.939	9.167	15.511	8.689	7.997	9.532
Estim. σ^2^ (time ^1^)	0.294	10.542	19.097	−0.258	6.113	5.580	0.061	0.103	0.391
Estim. σ^2^ (PwA)	148.985	162.843	146.692	175.167	140.340	149.500	168.381	167.186	151.898
Estim. σ^2^ (residual)	27.918	12.151	27.038	8.939	9.167	15.511	8.689	7.997	9.532
Pearson r	0.842	0.931	0.844	0.952	0.944	0.914	0.952	0.956	0.947
ICC (consistency) ^§^	0.842	0.931	0.844	0.951	0.939	0.906	0.951	0.954	0.941
ICC (agreement) ^§^	0.841	0.878	0.761	0.953	0.902	0.876	0.951	0.954	0.939

^1^ Respectively, MS (version) for the analysis of consistency among both versions of the ANELT. ^#^ From a repeated-measures one-factorial ANOVA with 2-level factor “time”, MS: mean squares, Estim. σ^2^: estimated variance component, ^§^ ICC (consistency) = ICC (3,1), ICC (agreement) = ICC (2,1), as defined by Shrout and Fleiss.

**Table 6:** Pearson correlation between ANELT communication scores and AAT spontaneous speech ratings and subtest T-scores, including the profile level estimate (reliability weighted average of subtest T-scores).

Correlation	T1		T2		T3		Diff. T3–T1	
AAT × ANELT	V1	V2	V1	V2	V1	V2	V1	V2
COM	0.740 ***	0.734 ***	0.742 ***	0.769 ***	0.832 ***	0.830 ***	0.009	−0.183
ART	0.129	0.149	0.105	0.044	0.296	0.273	0.198	0.132
AUT	0.575 **	0.639 **	0.601 **	0.574 **	0.606 ***	0.612 ***	0.097	−0.081
SEM	0.510 **	0.545 *	0.517 *	0.510 **	0.633 ***	0.627 ***	0.032	−0.096
PHO	0.402 *	0.317	0.340	0.300	0.540 **	0.477 *	0.029	−0.115
SYM	0.681 ***	0.667 ***	0.640 ***	0.583 **	0.839 ***	0.766 ***	−0.111	−0.191
Profile level	0.898 ***	0.855 ***	0.839 ***	0.839 ***	0.827 ***	0.850 ***	−0.163	−0.313
Token Test	0.811 ***	0.801 ***	0.812 ***	0.795 ***	0.727 ***	0.777 ***	−0.123	−0.264
Repetition	0.622 ***	0.557 **	0.536 **	0.544 **	0.681 ***	0.633 ***	−0.165	−0.309
Written language	0.863 ***	0.823 ***	0.747 ***	0.701 ***	0.809 ***	0.796 ***	−0.159	−0.253
Naming	0.851 ***	0.900 ***	0.868 ***	0.837 ***	0.854 ***	0.848 ***	−0.127	−0.249
Comprehension	0.672 ***	0.728 ***	0.719 ***	0.879 ***	0.744 ***	0.808 ***	0.028	−0.125

* *p* < 0.05, ** *p* < 0.01, *** *p* < 0.001.

## Data Availability

The original contributions presented in the study are included in the article, in Appendix A, and (as indicated) in [38]; further inquiries may be directed to the corresponding author.

## Abbreviations

The following abbreviations are used in this manuscript:

AAT	Aachen Aphasia Test
ANELT	Amsterdam–Nijmegen Everyday Language Test
ASHA-FACS	American Speech-Language-Hearing Association Functional Assessment of Communication Skills for Adults
BIAS-R	Bielefeld Aphasia Screening-Reha
CETI	Communicative Effectiveness Index
FIM	functional independence measure
FPC	frontopolar cortex
IFG	inferior frontal gyrus
M1	primary motor cortex
PwA	person with aphasia
SLT	speech and language therapy
SLP	speech and language pathologist
tDCS	transcranial direct current stimulation

## Appendix A. Additional Tables

**Table A1:** Communication data for AAT/ANELT sample at all three measurements, T1, T2, and T3.

Raw Scores (Max. 50)	ANELT—Version 1			ANELT—Version 2		
PwA	T1	T2	T3	T1	T2	T3
1 ^§^	40	28	50			
2	37	34	42	29	26	36
3 ^§^	16	23	29		20	27
4	50	43	48	49	45	48
5	36	44	45	39	45	46
6	50	50	50	50	50	50
7	33	38	40	43	40	37
8	39	40	44	44	37	37
9	37	49	49	37	44	43
10 ^#§^			12			13
11	11	15	16	13	18	20
12	19	12	14	14	14	16
13	44	47	50	43	46	44
14 ^§^	10	32	41		36	46
15 ^§^	13	25	28		24	31
16	17	17	23	16	20	23
17	41	38	47	39	40	42
18	14	15	17	14	17	21
19	48	49	47	44	47	48
20	24	12	18	20	15	14
21	22	23	25	27	18	24
22	14	17	17	12	15	17
23	16	12	18	12	12	27
24	27	31	41	24	26	31
25	11	10	19	11	12	16
26	26	25	33	19	24	28
27	32	41	41	28	28	37

^#^ PwA not included in analyses of ANELT Version 1; PwA identifier corresponds to Table A4 in [38]. ^§^ PwA not included in analyses of ANELT Version 2; PwA identifier corresponds to Table A5 in (Rubi-Fessen et al., 2024) [38].

**Table A2:** Communication data for BIAS-R/Scenario Test sample at all three measurements, T1, T2, and T3.

	Quantitative Raw Score (Max. 54)			Propositions Transmitted (Max. 24)		
PwA	T1	T2	T3	T1	T2	T3
1 ^#^ (28) ^§^						
2 ^#^ (29)		14	10		3	1
3 (30)	15	13	16	1	0	0
4 (31)	27	25	30	7	5	12
5 (32)	11	11	17	0	0	1
6 (33)	27	32	45	7	11	19
7 (34)	16	29	43	2	11	19
8 (35)	15	13	31	1	0	11
9 (36)	11	14	22	0	3	5
10 (37)	14	12	18	0	0	1

^#^ PwA not included in analyses of the Scenario Test; PwA identifier corresponds to Table A5 in [38]. ^§^ PwA identifier in brackets corresponds also to identifiers 28–37 from Table A3 in [38], reporting individual participant characteristics.
